# When discordant insulin and C-peptide levels lead to a medical diagnosis in a patient with transient hypoglycemia: Varying degrees of interference of insulin-antibody complexes on three insulin immunoassays

**DOI:** 10.1016/j.heliyon.2024.e34009

**Published:** 2024-07-02

**Authors:** Jordan Teoli, Karim Chikh, Ryme Jouini-Bouhamri, Sybil Charriere, Nicole Fabien, Véronique Raverot

**Affiliations:** a Laboratoire de Biologie Médicale Multisites, Service de Biochimie et Biologie Moléculaire, UM Biologie Endocrinienne, Centre de Biologie et Pathologie Est, Hospices Civils de Lyon, Bron, France; b Université de Lyon, Université Claude Bernard Lyon 1, Lyon, France; c Institut Cellule Souche et Cerveau (SBRI), Unité INSERM 1208, Centre de Recherche INSERM, Bron, France; d Laboratoire de biologie Médicale Multisites, Service de Biochimie et Biologie Moléculaire, UM Biologie Endocrinienne, Centre de Biologie et Pathologie Sud, Hospices Civils de Lyon, Pierre-Bénite, France; e Laboratoire CarMen, UMR INSERM U1060/INRAE U1397, Université Claude Bernard Lyon 1, Pierre Bénite, France; f Fédération d'Endocrinologie, Maladies Métaboliques, Diabète, et Nutrition, Hôpital Louis Pradel, Hospices Civils de Lyon, Bron, France; g Service d'immunologie, Centre de Biologie et Pathologie Sud, Hospices Civils de Lyon, Pierre-Bénite, France

**Keywords:** Case report, Insulin autoimmune syndrome, Hyperinsulinism, Immunoassay, Assay interference

## Abstract

**Background:**

Determining the cause of hypoglycemia partly relies on blood insulin and C-peptide assays. Although the pancreatic secretion of these peptides is equimolar, discrepancies in their concentrations may occur.

**Case presentation:**

We report the case of a 73-year-old woman with type 2 diabetes mellitus (T2DM) and a history of gastric bypass. The T2DM was initially treated with insulin analogs, which were interrupted due to transient hypoglycemia episodes three years before hospitalization in our endocrinology department. During this hospitalization, the most common etiologies of hypoglycemia were excluded. Fasting insulin level was high (190 mIU/L, reference values (RV): 5–25) on Architect i2000 (an assay recognizing insulin analogs) despite normal blood C-peptide (4.5 μg/L, RV: 0.8–5.2) and slight hypoglycemia (4.5 mmol/L, RV: 4.6–6.1). Insulin level using the Elecsys assay (an assay with low sensitivity to insulin analogs) was very high (>1000 mIU/L, RV: 2.6–24.9). This pattern was observed on several samples, including some taken during a fasting test. Insulin level was only slightly increased using the Mercodia iso-insulin ELISA kit (an assay recognizing insulin analogs). These results excluded an exogenous insulin intake and were suggestive of an interference on insulin assays. To explore the latter possibility, free anti-insulin antibodies were measured and found strongly positive. The presence of interfering insulin-antibody complexes was further investigated using gel filtration chromatography, polyethylene glycol precipitation, and dilution test. Based on these findings, an insulin autoimmune syndrome (IAS) was suspected to cause the hypoglycemic episodes observed.

**Conclusion:**

Although a discrepancy between blood insulin and C-peptide levels points to insulin analog intake, IAS should also be considered, particularly in a patient with transient hypoglycemia. IAS is characterized by the presence of insulin-antibody complexes, which can induce varying degrees of interference on insulin immunoassays and may lead to discordant insulin and C-peptide levels according to the insulin immunoassay used.

## Highlights

• Insulin autoimmune syndrome should be suspected when insulin/C-peptide ratio >1.
• The sensitivity to antibody-bound insulin varies among different insulin assays.
• Measurement using Mercodia iso-insulin assay appears to reflect free insulin levels.
• PEG-precipitation interpretation is complicated by serial dilution non-linearity.

## 1 Introduction

Determining the cause of hypoglycemia partly relies on blood insulin and C-peptide assays [1,2]. Although the secretion of these peptides by pancreatic beta cells is equimolar, the molar ratio of insulin/C-peptide in blood is physiologically far below 1 (approximately 0.1–0.2) [[3], [4], [5]]. This is due to differences in insulin and C-peptide metabolism. Insulin is mainly metabolized by the liver whereas C-peptide is mainly metabolized by the kidney [4]. Moreover, insulin clearance is faster than that of C-peptide resulting in a shorter blood half-life for insulin (5–10 minutes) than for C-peptide (30–35 minutes) [3,4].

In some cases of hypoglycemia, a molar insulin/C-peptide ratio greater than 1 may occur. This may be due to exogenous insulin intake, which may cross-react with certain insulin assays, leading to high insulin blood concentration measurements. In this situation, the endogenous insulin secretion is slowed, which is revealed by a decrease in C-peptide concentration, and results in an insulin/C-peptide ratio greater than 1 [4,6]. Another cause of hypoglycemia with insulin and C-peptide level discrepancy is insulin autoimmune syndrome (IAS) [[1], [2], [3],^6^].

IAS is characterized by the presence of serum anti-insulin antibodies (AIA), which capture endogenous insulin, preventing its action, and then release it independently of blood glucose leading to hypoglycemia, sometimes severe [2,6]. The antibody-bound insulin has a reduced clearance and may react with some insulin assays, resulting in higher circulating insulin concentration and an increased insulin/C-peptide ratio, possibly greater than 1 [3,[6], [7], [8]].

We report the case of a patient with a 3-year history of unexplained hypoglycemic episodes in whom the insulin/C-peptide ratio greater than 1 allowed us to suspect IAS. The presentation of the case herein is an opportunity to extend knowledge on IAS and to compare the impact of the presence of AIA on three different insulin immunoassays. To our knowledge, this is the first time that the impact of these AIA on the insulin levels detected by the Architect i2000 and Mercodia iso-insulin ELISA assays is investigated using polyethylene glycol (PEG) precipitation with or without prior acidification. A subsequent dilution test was also performed using Mercodia iso-insulin ELISA assay.

## 2 Case presentation

The patient described herein signed an informed consent for the publication of her anonymized data. The study was reviewed and approved by the scientific and ethics committee of the *Hospices Civils de Lyon*, with the approval number 23–5202 (approved on July 04, 2023).

### 2.1 Patient information and history

We report the case of a 73-year-old Caucasian woman with type 2 diabetes mellitus, hospitalized in the endocrinology department (Lyon, France) on March 23, 2023 for the investigation of hypoglycemic episodes despite the discontinuation of hypoglycemic treatments.

**Fig. 1:**
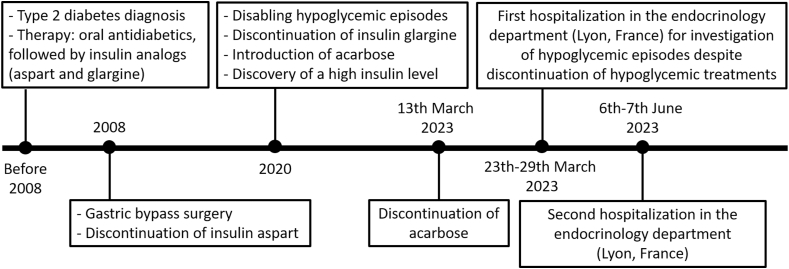
Patient's history.

The patient presented with type 2 diabetes mellitus for at least 15 years and was treated first with oral antidiabetics and then insulin analog therapy (insulin aspart and insulin glargine). In 2008, the patient had a gastric bypass at 58 years old due to obesity with sleep apnea. Insulin aspart was stopped within the first year following the surgery. Insulin glargine was stopped 12 years later, in 2020, due to disabling hypoglycemic episodes associated with sweating, tremors, and a feeling of tiredness. Insulin glargine was replaced by an acarbose treatment and hypoglycemia was explored. A high insulin level was found, which led to suspect an insulinoma, but no pancreatic lesions were found on three abdominal CT scans performed over a four-year period (2020, 2021, 2023). Acarbose treatment was stopped 10 days before the patient's hospitalization in the endocrinology department (Fig. 1).

At admission (March 23, 2023), the patient had no other treatment except occasional bromazepam. The patient's weight was 87 kg with a BMI of 33 kg/m^2^. The patient had stopped smoking for 3 years and drank alcohol occasionally. There was no personal nor familial history of autoimmune disease. Her parents and one of her brothers had type 2 diabetes mellitus.

### 2.2 Diagnostic assessment during hospitalization in the endocrinology department (March 23 to March 29, 2023)

Episodes of late postprandial (away from mealtimes, e.g. mid-afternoon or early evening) capillary hypoglycemia (minimum 1.7 mmol/L) were observed during the patient's hospitalization in the endocrinology department, even though the patient had a habit of pre-emptively eating (cereal bars every 2 h) to prevent hypoglycemia or when she felt adrenergic hypoglycemic symptoms. HbA1c was at 6.1 %.

The most common etiologies of hypoglycemia were ruled out as follows: the absence of digestive disorders was not in favor of dumping syndrome; blood measurements did not show plasma cortisol deficiency (8 a.m. cortisolemia at 446 nmol/L [reference values-RV: 145–535], above the cut-off previously described by our team to exclude adrenal insufficiency [9], with ACTH at 53 ng/L [RV: 7–63]) or serum IGF1 (147 μg/L [RV: 37–165]) deficiency nor renal deficiency (creatininemia at 62 μmol/L [RV: 49–90]); liver function tests (plasma alanine aminotransferase, aspartate aminotransferase, total bilirubin, alkaline phosphatase, gamma-glutamyl transferase) were normal. The patient then underwent a 72-h fasting test (March 24 to March 27, 2023) with glycemia, insulinemia, and blood C-peptide measurements, controlled by positive capillary and urinary ketone bodies. The results were evocative of a hyperinsulinemic etiology of the hypoglycemic episodes (Table 1). During this test, insulinemia was initially assessed by an assay recognizing insulin analogs (Architect i2000, Abbott, North Chicago, Illinois, USA; see Table 2 for insulin assay characteristics) [[10], [11], [12]]. Therefore, the presence of a very high fasting insulinemia (190 mIU/L, RV: 5–25) in contrast to a normal blood C-peptide level (4.5 μg/L on Architect i2000, RV: 0.8–5.2; Table 1) raised the question of hidden injections of insulin analogs not admitted by the patient (exogenous hyperinsulinemia). This hypothesis however, was ruled out for four reasons: i) because the glycemia was not as low as could be expected (4.5 mmol/L for a reference range of 4.6–6.1) given the hyperinsulinemia at 190 mIU/L at day (D) 1 (March 24, 2023), ii) because the C-peptide concentration should be decreased in such a situation, iii) because the hyperinsulinemia was secondarily confirmed using an assay with low sensitivity to insulin analogs (Elecsys insulin assay on Cobas e411, Roche Diagnostics, Basel, Switzerland; Table 1, Table 2) [11,12], and iv) because the insulinemia was not as high as expected using a third assay recognizing insulin analogs (iso-insulin ELISA kit from Mercodia, Uppsala, Sweden; Table 1, Table 2) [11,12]. The exclusion of exogenous hyperinsulinemia, with the addition of a normal C-peptide concentration, guided the diagnosis toward an endogenous hyperinsulinemia. The absence of hypoglycemia <2.5 mmol/L during the fasting test and the absence of pancreatic lesions on the three abdominal CT scans performed prior to her hospitalization were not in favor of an insulinoma. Furthermore, the intake of insulin secretagogues (repaglinide and sulphonylurea [glibenclamide, gliclazide, glimepiride, glipizide]) was investigated on a D5 sample (March 28, 2023) using in-house liquid chromatography coupled with tandem mass spectrometry and was negative. These findings, associated with an elevated insulin/C-peptide molar ratio (sometimes >1 according to the sample and assay considered), finally guided the diagnosis toward an IAS. Therefore, free AIA measurement was performed using a radioimmunoassay detecting all immunoglobulin classes and subclasses (DiaSource Diagnostics, Ottignies-Louvain-la-Neuve, Belgium). The AIA level was extremely high (about 90 %, RV <8.2 %) on several samples (Table 1). The presence of insulin-antibody complexes was then confirmed retrospectively using the two strategies below.

**Table 1:** Laboratory investigations during the two hospitalizations in the endocrinology department.

Date (since first entry in the endocrinology department) and sample characteristics	Plasmatic glycemia (4.6–6.1 mmol/L)	Insulinemia on EDTA plasma (Architect i2000 insulin assay) (5–25 mIU/L)	Insulinemia on serum (Elecsys insulin assay, Cobas e411) (2.6–24.9 mIU/L)	Insulinemia on serum (Mercodia iso-insulin ELISA kit) (2–25 mIU/L)	C-peptide on EDTA plasma (Architect i2000 C-peptide assay) (0.8–5.2 μg/L)	Free AIA on serum (<8.2 %)
D1 - 7:30 a.m. (March 24, 2023) Fasting test Non-hemolyzed sample	4.5 ↘	190 ↗↗ (0.91)^a^	>1000 ↗↗↗ (>4.78)^a^ 1813 ↗↗↗ (dilution 1/10)	NA	4.5	89.35 % ↗↗↗
D2 - 8:00 a.m. (March 25, 2023) Fasting test Non-hemolyzed sample	4.0 ↘	129 ↗↗ (1.21)^a^	>1000 ↗↗↗ (>9.36)^a^ 1426 ↗↗↗ (dilution 1/10)	NA	2.3	NA
D3 - 8:10 a.m. (March 26, 2023) Fasting test Non-hemolyzed sample	3.4 ↘	92 ↗↗ (0.99)^a^	720.7 ↗↗↗ (7.76)^a^ 934 ↗↗↗ (dilution 1/10)	NA	2.0	NA
D4 - 8:00 a.m. (March 27, 2023) Fasting test Non-hemolyzed sample	2.9 ↘	86 ↗↗ (1.32)^a^	738.7 ↗↗↗ (11.36)^a^ 811 ↗↗↗ (dilution 1/10)	NA	1.4	NA
D5 - 9:00 a.m. (March 28, 2023) Non-hemolyzed sample	NA	193 ↗↗	>1000 ↗↗↗ 2782 ↗↗↗ (dilution 1/10)	27.5 ↗	NA	89.98 % ↗↗↗
D6 - 8:40 a.m. (March 29, 2023)	NA	NA	>1000 ↗↗↗	28.0 ↗	NA	90.08 % ↗↗↗
D76–8:15 a.m. (June 7, 2023) Non-hemolyzed sample	4.7	273 ↗↗↗ (1.01)^a^	>1000 ↗↗↗ (>3.71)^a^	27.0 ↗ (0.10)^a^	5.8 ↗	87.52 % ↗↗↗
D76–11:20 a.m. (June 7, 2023) Non-hemolyzed sample	NA	269 ↗↗↗	>1000 ↗↗↗	54.6 ↗	NA	NA

The assays and their reference range are detailed in brackets in the column headings. C-peptide assay shows <0.01 % of cross-reactivity with insulin (supplier data). Please note that the AIA assays were performed on D1 and D5 samples having undergone a −20°c freeze-thaw cycle.

AIA: anti-insulin antibodies; D: day; N: normal; NA: not available; PEG: polyethylene glycol; ↘ or ↗: decreased or increased, respectively, when compared to reference ranges.

a (.) = insulin (pmol/L)/C-peptide (pmol/L) ratio. Conversion factor used (data from Architect i2000 assays): insulin mIU/L x 7.175 = insulin pmol/L; C-peptide μg/L x 333.33 = C-peptide pmol/L.

**Table 2:** Insulin assay characteristics (supplier data unless otherwise specified).

	Architect i2000 insulin assay (Abbott)	Elecsys insulin assay on Cobas e411 (Roche)	Iso-insulin ELISA assay (Mercodia)
Cross-reactivity with recombinant human insulins and insulin analogs	Assay sensitive to recombinant human insulins and insulin analogs [[10], [11], [12]]. According to Moriyama et al. [10]: Insulin lispro (10 mIU/L) 110 %, Insulin lispro (100 mIU/L) 100 %, Insulin aspart (10 mIU/L) 76 %, Insulin aspart (100 mIU/L) 75 %, Insulin glargine (10 mIU/L) 105 %, Insulin glargine (100 mIU/L) 83 %, C-peptide (1.10^7^ ng/L) < 0.00001 %, Proinsulin (1.10^6^ ng/L) < 0.005 %. According to Violin et al. [11]: Insulin lispro 101 %, Insulin aspart (NovoRapid®) 82 %, Insulin aspart (NovoMix® 50) 30 %, Insulin detemir 139 %, Insulin glargine 110 %, Insulin glulisine 8.7 %, Insulin degludec 33 %.	Assay sensitive to recombinant human insulins but absent or very low sensitivity to insulin analogs [11,12], except for insulin glargine metabolite M1 (cross-reactivity around 22 % [24]). From supplier and [25]: C-peptide (100 ng/ml) not detectable, Human proinsulin (1000 ng/ml) 0.05 %, Proinsulin des (31–32) 20 %, Proinsulin des (64–65) 0.1 %.	Assay sensitive to recombinant human insulins and insulin analogs [11,12]. From supplier: Insulin lispro 112 %, Insulin aspart 100 %, Insulin detemir 28 %, Insulin glargine 58 %, Insulin glargine M1 47 %, Insulin glargine M2 32 %, Insulin glulisine 123 %, C-peptide <0.1 %, Proinsulin 54 %, Proinsulin des (31–32) 58 %, Proinsulin split (32–33) 56 %, Proinsulin des (64–65) 66 %, Proinsulin split (65–66) 78 %.
Assay principle	Chemiluminescent microparticle immunoassay Sandwich one step	Electrochemiluminescence immunoassay Sandwich one step	ELISA Sandwich one step
Dilution factor of plasma/serum sample induced by addition of reagents during assay	5.2	8.5 [8]	3
Incubation time in the presence of capture and detection antibodies	25 minutes	9 minutes	1 hour
Incubation temperature in the presence of capture and detection antibodies	37°c	37°c [8]	Room temperature (18–25°c)
Hook effect	Absent at least until 30000 mIU/L [10]	Absent at least until 20000 mIU/L	Absent at least until 2000 mIU/L
Upper limit of linearity	300 mIU/L	1000 mIU/L	100 mIU/L
Lower limit of measurement range	1 mIU/L (limit of detection)	0.2 mIU/L (limit of detection)	3 mIU/L (first calibration point)

### 2.3 Laboratory investigations for insulin-antibody complexes

First, a gel filtration chromatography (procedure and system derived from Ref. [13]) was performed on the D76–8:15 a.m. sample having undergone a −20°c freeze-thaw cycle (Table 1). To that end, 1 ml of plasma was mixed with 200 μl of a solution of Blue Dextran (10 mg/ml in phosphate-buffered saline, PBS) and deposited on a chromatography column 1.5 × 50 cm x 5 (ref 290203, Dutscher, Bernolsheim, France) filled with Sephacryl® 200-HR (ref S200HR, Sigma-Aldrich, Saint-Louis, Missouri, USA). PBS was used as elution buffer and pumped through the column using a MINIPULS 3 peristaltic pump (Gilson, Wisconsin, USA). The FC 203B fraction collector (Gilson, Madison, Wisconsin, USA) was used to collect 1.5 ml fractions exiting from the column progressively (4 min per 1.5 ml fraction, flow rate of 0.4 ml/min). Then, insulinemia was measured in each fraction using the Architect i2000 assay. This procedure highlighted the presence of an insulin-containing compound of higher molecular weight (>100 kDa) than the insulin monomer molecular weight (5.8 kDa).

**Fig. 2:**
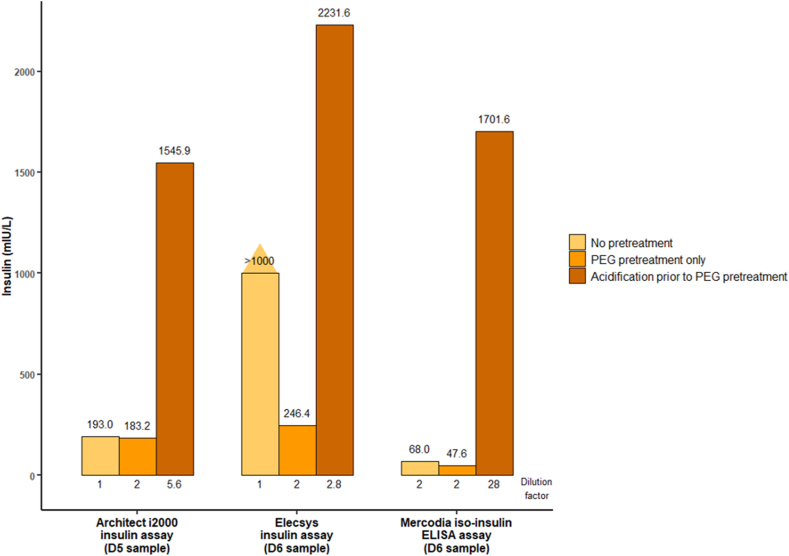
**Polyethylene glycol precipitation tests with or without prior acidification.** The figure compares insulin levels obtained with several assays with different pretreatments. Insulin levels measured after PEG pretreatment only is designed to represent the free insulin fraction. Insulin levels measured after acidification prior to PEG pretreatment is designed to represent total insulin. After PEG pretreatment only, insulin concentration reduction, calculated as (insulin measured with no pretreatment – free insulin) x 100/insulin measured with no pretreatment, is 5.1 % using the Architect i2000 insulin assay on the D5 sample and insulin concentration is reduced by >75.4 % and 30.0 % using the Elecsys insulin and the Mercodia iso-insulin ELISA assays, respectively, on the D6 sample. The antibody-bound insulin fraction, estimated by (total insulin – free insulin) x 100/total insulin, is equal to 88.1 %, 89.0 %, and 97.2 % using the Architect i2000 insulin, Elecsys insulin, and Mercodia iso-insulin ELISA assays, respectively. Insulin concentrations (y-axis) were calculated by taking into account the dilution factor (if any). The dilution factor is indicated under each bar of the graph and corresponds to the total dilution factor of the sample, i.e. including dilution by PEG solution, dilution by acidification protocol, and dilution by the Architect Insulin Calibrator A or the calibrator 0 from the Mercodia iso-insulin ELISA kit when used. When possible, insulin values obtained from a same dilution factor were displayed on the plot for an optimal comparison. When this was not possible, insulin values obtained from the nearest dilution factor were displayed. Note that these tests were performed retrospectively during the diagnostic approach on samples having undergone a −20°c freeze-thaw cycle.

Secondly, we adapted the protocol described by Hattori et al. [14] to estimate the antibody-bound insulin fraction using the Architect i2000 insulin, the Elecsys insulin (Cobas e411), and the Mercodia iso-insulin ELISA assays. Three samples (D1 – 7:30 a.m., D5 - 9:00 a.m., and D6 - 8:40 a.m.) underwent the following procedure, in which 2 mixes were prepared in parallel for each of the three samples. In the first mix (PEG pretreatment only), 100 μl of a 25 % (m/v) PEG 6000 (Merck, Darmstadt, Germany) solution prepared in PBS were added to 100 μl of plasma. After shaking and waiting 10 minutes at room temperature, the mix was centrifuged (3700×*g*, +6°c, 15 minutes). Insulinemia was measured in the supernatant and considered as free insulin after taking into account the dilution factor of 2. In the second mix (acidification prior to PEG pretreatment), 20 μl of hydrochloric acid 1 N were added to 100 μl of plasma, and the mixture was left for 1 hour at room temperature. Next, 140 μl of the PEG solution 25 % (m/v) and 20 μl of NaOH 1 N were added. After shaking and waiting 10 minutes at room temperature, the mix was centrifuged (3700×*g*, +6°c, 15 minutes). Insulinemia was measured in the supernatant and considered as total insulin after taking into account the dilution factor of 2.8 (Fig. 2). When insulinemia was over the upper limit of linearity of the assay, further dilutions were performed using the Architect Insulin Calibrator A or the calibrator 0 from the Mercodia iso-insulin ELISA kit. The antibody-bound insulin fraction, estimated by (total insulin – free insulin) x 100/total insulin, was equal to 88.1 %, 89.0 %, and 97.2 % using the Architect i2000 insulin, the Elecsys insulin (Cobas e411), and the Mercodia iso-insulin ELISA assays, respectively (Fig. 2). After PEG pretreatment only, insulin concentration reduction, calculated as (insulin measured with no pretreatment – free insulin) x 100/insulin measured with no pretreatment, was 24.2 % (190.0 mIU/L without pretreatment and 144.0 mIU/L after PEG pretreatment only) and 5.1 % (Fig. 2) using the Architect i2000 insulin assay on D1 and D5 samples, respectively. After PEG pretreatment only, insulin concentration on the D6 sample was reduced by >75.4 % and 30.0 % using the Elecsys insulin (Cobas e411) and the Mercodia iso-insulin ELISA assays, respectively (Fig. 2).

**Fig. 3:**
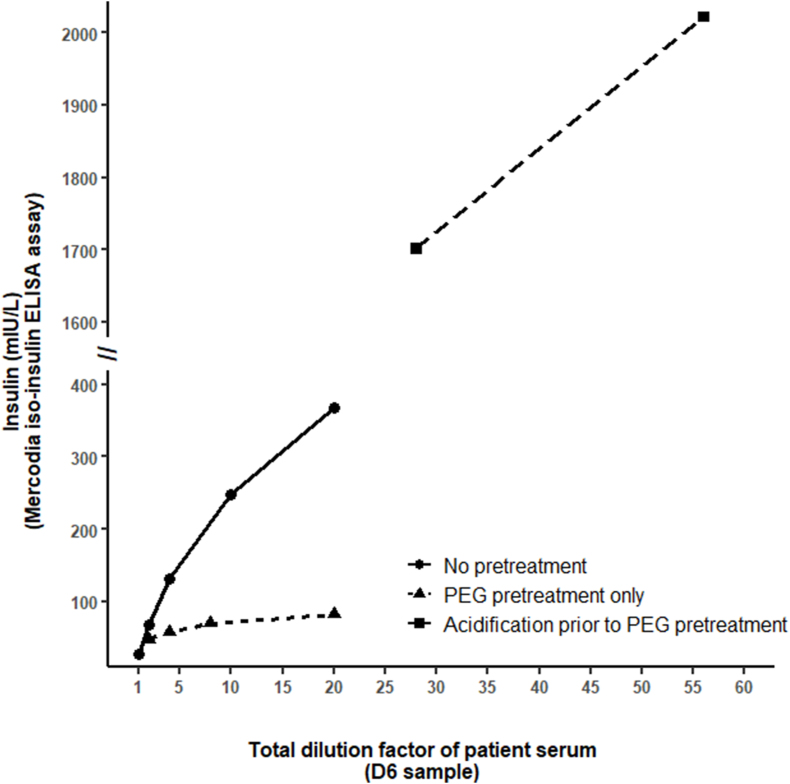
**Insulin measurement using the Mercodia iso-insulin ELISA assay on different dilutions and pretreatments of the D6 sample.** The figure shows a progressive increase in the insulin measured values when the dilution factor increases (non-linearity of insulin concentration measurement with the dilution factor) in the three situations tested. In the “PEG pretreatment only” situation, this dilution effect persists but is strongly reduced. The total dilution factor of the sample includes dilution by the calibrator 0 from the Mercodia iso-insulin ELISA kit and, when applicable, dilution by PEG solution and by acidification protocol. For some dilutions, values were below the lower limit of measurement range or under the upper limit of linearity and are not displayed on the figure: for dilution factor 40, insulin concentration after PEG pretreatment only was <80 mIU/L; for dilution factors 2.8, 5.6, and 11.2, insulin concentrations after acidification prior to PEG pretreatment were >280, >560, >1120, respectively. Note that these tests were performed retrospectively during the diagnostic approach on the D6 sample having undergone a −20°c freeze-thaw cycle.

Furthermore, to exclude a hook effect on insulin measurement using the Mercodia iso-insulin ELISA kit, a dilution test using the calibrator 0 from this kit was performed on the D6 - 8:40 a.m. sample. A progressive increase in the insulin measured values was observed when the dilution factor increased (Fig. 3). A dilution test was also performed on the supernatant of the D6 - 8:40 a.m. sample obtained either after PEG pretreatment only or after acidification prior to PEG pretreatment. The progressive increase in the insulin measured values according to the dilution factor increase was less important after PEG pretreatment only compared to no pretreatment (Fig. 3).

### 2.4 Extension assessment

The patient complained of joint pains in the fingers and shoulders, with morning stiffness. An extension assessment was performed to ensure that there were no other associated autoimmune diseases. Antinuclear antibodies, anti-double-stranded DNA antibodies, antichromatin antibodies, anti-neutrophil cytoplasm antibodies, anti-GAD65 antibodies, anti-IA2 antibodies, anti-ZnT8 antibodies, anti-thyroperoxydase antibodies, anti-TSH receptor antibodies, IgA anti-transglutaminase antibodies, and anti-cyclic citrullinated peptide antibodies were negative. Only anti-parietal cell antibodies were found positive without clinical signs nor macrocytic anemia. Serum protein electrophoresis did not reveal monoclonal gammopathy. Immunoglobulin (Ig) A level was slightly increased (4.24 g/L, RV: 0.78–4.11) and Ig M level slightly decreased (0.35 g/L, RV: 0.40–2.80). Ig G and rheumatoid factor levels were normal.

### 2.5 Therapeutic intervention and follow-up

When leaving the endocrinology department (March 29, 2023), the patient was advised to stop eating pre-emptively regularly during the day and to instead assess capillary glycemia and take snacks, consisting preferably of slow carbohydrates with dairy products rather than fast carbohydrates, only if the result was below 2.8 mmol/L. The patient was also advised to eat complete meals, consisting of complex carbohydrates, fiber, dairy products, protein, and fat, three times a day. Two months later, the patient was hospitalized again in the endocrinology department (June 6 to June 7, 2023) to re-evaluate her glycemic state (Fig. 1). The patient reported an absence of hypoglycemic episode since her last hospitalization (2-month period). However, an asymptomatic capillary hypoglycemia was detected (2.8 mmol/L at 11 p.m. on June 6, 2023) and the hyperinsulinemia associated with strongly positive AIA pattern remained on new samples taken in the endocrinology department (D76 samples on June 7, 2023; Table 1). A continuous glucose monitoring (FreeStyle Libre Pro) was then introduced. During the following two weeks after the second hospitalization (June 7 to June 23, 2023), low glucose levels (between 54 and 69 mg/dl, target range: 70–180) were found during 4 % (objective <4 %) of the time, especially in the afternoons and evenings; no very low glucose level (<54 mg/dl) was observed (objective <1 %). Glucose level was high (>180 mg/dl) during 5 % (objective <25 %) of the time. The patient was reminded to eat low carbohydrates at every meal and to not skip the morning meal as she was accustomed to doing. Acarbose and insulin analogs were not re-introduced and no other therapy was initiated since the glycemic situation improved under dietary advice.

## 3 Discussion

We report the case of a patient with type 2 diabetes mellitus and a history of gastric bypass surgery who presented with hypoglycemic episodes despite the disruption of all oral antidiabetic drugs and insulin analog therapy. The patient had no known autoimmune disease. Hypoglycemia etiology was investigated using the structured clinical approach recently proposed by Cappellani et al. and Haverkamp et al. [1,2]. After the exclusion of exogenous insulin intake, the hyperinsulinemia with conserved C-peptide level, in a hypoglycemic context, associated with strongly positive free AIA and the presence of insulin-antibody complexes, were evocative of an autoimmune origin of the hypoglycemia. The diagnosis of IAS, possibly induced by previous insulin analog intake (exogenous IAS [15] or non-classical form of IAS [6]), was thus very likely, although it could not be fully ascertained. Indeed, a dumping syndrome in the context of gastric bypass surgery could not be completely excluded as a contributor to hypoglycemia episodes, although it was unlikely considering the absence of digestive disorders. Moreover, in IAS, hypoglycemia is known to occur in the presence of low-affinity/high-binding capacity AIA [6]; unfortunately, the affinity and binding capacity of AIA were not explored herein. Given the suspicion of IAS, the interference of AIA on insulin immunoassays was then further explored.

IAS is a rare endocrine disorder, even rarer in western countries [6]. The context of IAS occurrence in the patient herein (type 2 diabetes mellitus with a history of oral antidiabetics and insulin analog treatment), her laboratory anomalies (absence of severe hypoglycemia during the 72-h fasting test, late postprandial hypoglycemia, high insulinemia with insulin/C-peptide ratio near or above 1, positive AIA), and the decrease in hypoglycemia episode frequency under dietary advice despite the persistence of strongly positive AIA two months after, are in line with data presented in the review of 795 IAS cases [6]. AIA can persist several years after the discontinuation of the insulin analogs involved in their occurrence [6,14,[16], [17], [18]]. Some authors have also suggested that endogenous insulin can support antibody formation after the interruption of insulin therapy [16,19]. Although the use of therapies aiming to reduce insulin secretion (diazoxide, octreotide) or AIA levels (immunosuppressive drugs such as glucocorticoids) or delay intestinal carbohydrate absorption (acarbose) have been reported in IAS [1,2,6], such therapies were not considered in the present case because of the improvement in the glycemic situation under dietary advice.

The findings presented herein show that different insulin immunoassays may react with antibody-bound insulin with varying degrees of sensitivity: Elecsys insulin assay on Cobas e411 showed the highest sensitivity (i.e. value approaching total insulin), Mercodia iso-insulin ELISA kit had the lowest sensitivity (i.e. value approaching free insulin), and Architect i2000 insulin assay had an in-between sensitivity. Discrepancies in insulin concentration between assays were found in a patient with AIA using Modular analytic E170 (Roche), Unicel DxI 800 (Beckman-Coulter), and Bi-Insulin IRMA (CIS bio) insulin assays [20]. In the same way, Sapin et al. showed that Elecsys insulin assay (Roche) was more sensitive to the presence of antibody-bound insulin than the manual radioisotopic Bi-Insulin IRMA assay from Bio-Rad [8]. Again, in a patient with AIA, discordant results for insulin measurement were obtained using ADVIA Centaur® (Siemens), Immulite® 2000 (Siemens), LIAISON® XL (DiaSorin), AutoDELFIA® (PerkinElmer), and Access 2 (Beckman Coulter) assays [21]. There are several explanations for these discrepancies in insulin concentration measurement according to the assay [8,20,21]. First, the insulin epitopes targeted by the assay antibodies might differ from one assay to another; hence some assays can recognize antibody-bound insulin whereas others cannot. Moreover, the shift in balance between free and bound insulin is dependent on several factors that might differ from one assay to another, such as the sample dilution inherent to the assay chemistry, the duration and temperature applied during the incubation step in the presence of assay antibodies, and/or the affinity for insulin of the assay antibodies used. In the latter explanation, an assay using antibodies with a higher affinity for insulin than that of the autoantibody will result in a higher measured insulin concentration than an assay using antibodies with lower affinity.

In the present case, a progressive increase in insulin concentration according to the increase in dilution factor of the sample was found using the Mercodia iso-insulin ELISA and Elecsys insulin assays. Such an increase in insulin concentration measurement according to an increase in dilution factor was previously described using a panel of insulin assays different from the ones studied herein [21] and is likely explained by a modification of the equilibrium between free and bound insulin. After PEG pretreatment only, this dilution effect persisted but was strongly reduced, suggesting that insulin-antibody complexes were responsible for the interference in insulin measurement and that all insulin-antibody complexes were not eliminated. This latter point is also supported by the remaining high insulin concentration measured using Elecsys insulin assay after PEG pretreatment only. Of note, it has been reported that the proportion of PEG-precipitation fraction may vary according to the immunoglobulin class involved in the immune complex [22]. However, the immunoglobulin class involved in the insulin-antibody complexes herein was not determined.

The PEG precipitation ratio was low using the Architect i2000 insulin (24.2 % and 5.1 %) and Mercodia iso-insulin ELISA assays (30.0 %) compared to the ratio obtained with the Elecsys insulin assay (Cobas e411, >75.4 %). Once again, the PEG precipitation ratio varied according to the assay's sensitivity in reacting with antibody-bound insulin (fraction precipitated after PEG pretreatment). Furthermore, as discussed above, a progressive increase in measured insulin concentration occurred when the dilution factor increased. Therefore, the dilution induced by the PEG pretreament protocol, although small, may underestimate the PEG precipitation ratio by overestimating the free insulin fraction measured after PEG pretreatment. To avoid this issue, the plasma/serum used for initial insulin measurement should be diluted by the same factor as the PEG pretreated plasma/serum. This correction was made for the calculation of the PEG precipitation ratio using the Mercodia iso-insulin ELISA assay but not for the Architect i2000 insulin assay, whose calculated ratios were underestimated. This dilution effect on insulin measurement, and therefore on PEG precipitation ratio estimation, seemed to be particularly relevant for assays with low or moderate sensitivity to antibody-bound insulin. In contrast to the low PEG precipitation ratios obtained using the Architect i2000 insulin and Mercodia iso-insulin ELISA assays, the PEG precipitation ratio obtained using the Elecsys insulin assay was in line with what was described elsewhere using the same or different assays [7,8,[21], [22], [23]]. Sapin et al. divided the insulin concentration by 96 after PEG precipitation using the Elecsys insulin assay in a patient with approximately 80 % of free AIA [8]. Yuan et al. showed a strongly reduced insulin concentration after PEG precipitation (>89 %) in patients with IAS using ADVIA Centaur® XP (Siemens), although insulin concentration could remain above the reference range [7]. Church et al. also emphasized discrepancies between assays when using PEG precipitation due to an assay-dependent matrix effect [21].

Using acidification to dissociate bound-insulin from antibodies prior to a PEG pretreatment to precipitate antibodies, we found a huge increase in insulin concentration. Here again, the different dilution induced by the PEG pretreament only protocol and by the acidification prior to PEG pretreatment protocol may misestimate the antibody-bound insulin fraction since a progressive increase in insulin concentration measured occurred when the dilution factor increased. Nevertheless, the antibody-bound insulin fraction determined using our three assays were largely above the threshold of 27.8 % defined by Hattori et al. in 23 non-diabetic control patients using an enzyme immunoassay for suspecting the presence of AIA [8]. Using Elecsys insulin assay, Sapin et al. showed that insulin concentration measured after acidification prior to PEG precipitation (total insulin) can be more than twice as high than insulin concentration measured without any pretreatment (direct insulin) [8], in line with what was found herein.

A discrepancy between blood insulin and C-peptide levels should raise the suspicion of insulin analog intake, if the insulin dosage technique is sensitive to it. This discrepancy should also raise the suspicion of an interference on insulin immunoassays caused by the presence of insulin-antibody complexes, specifically when the C-peptide level is not very low, when the measured insulin levels are strikingly different according to the immunoassays used, and when there is no evidence of exogenous insulin intake. However, in the presence of insulin-antibody complexes, the measured insulin level may be normal, increased, or strongly increased depending on the assay's sensitivity to react with the complexes. For instance, if the Mercodia iso-insulin ELISA kit had been used as the first line insulin assay herein, the diagnosis of IAS in the patient may not have been suspected. These complexes can be highlighted by gel filtration chromatography and by insulin release after acidification prior to PEG pretreatment. However, the concentration measured after PEG pretreatment only must be interpreted carefully according to the assay's sensitivity to the insulin-antibody complexes and should ideally be compared to the concentration measured before PEG pretreatment on a sample diluted by the same factor. The present case report underlines the importance of a good knowledge regarding the insulin assays used, as well as discussions between clinicians and biologists in order to avoid misdiagnosis.

## Funding source

This research did not receive any specific grant from funding agencies in the public, commercial, or not-for-profit sectors.

## Data availability

Data will be made available on request.

## CRediT authorship contribution statement

**Jordan Teoli:** Writing – original draft, Visualization, Validation, Software, Resources, Methodology, Investigation, Formal analysis, Conceptualization. **Karim Chikh:** Writing – review & editing, Validation, Resources, Methodology, Investigation, Formal analysis. **Ryme Jouini-Bouhamri:** Writing – review & editing, Validation, Resources, Investigation, Conceptualization. **Sybil Charriere:** Writing – review & editing, Validation, Supervision. **Nicole Fabien:** Writing – review & editing, Validation, Resources, Methodology, Investigation, Formal analysis. **Véronique Raverot:** Writing – review & editing, Visualization, Validation, Supervision, Resources, Methodology, Investigation, Formal analysis, Conceptualization.

## Declaration of competing interest

The authors declare that they have no known competing financial interests or personal relationships that could have appeared to influence the work reported in this paper.
